# Health insurance in Ghana: evaluation of policy holders’ perceptions and factors influencing policy renewal in the Volta region

**DOI:** 10.1186/1475-9276-12-50

**Published:** 2013-07-03

**Authors:** Daniel Boateng, Dadson Awunyor-Vitor

**Affiliations:** 1 Department of community Health, Kwame Nkrumah University of Science and Technology, Kumasi, Ghana; 2 Department of Agricultural Economics, Agribusiness and Extension, Kwame Nkrumah University of Science and Technology, Kumasi, Ghana

**Keywords:** Health insurance, Ghana, Policy renewal, Volta region

## Abstract

**Background:**

Health insurance is an important mechanism that succors individuals, states and the nation at large. The purpose of this study was to assess individual’s attitude towards health insurance policy and the factors that influence respondents’ decision to renew their health insurance policy when it expires.

**Methods:**

This cross sectional study was conducted in the Volta region of Ghana. A total of 300 respondents were randomly sampled and interviewed for the study. Data was collected at the household level and analyzed with STATA software. Descriptive statistics was used to assess the demographic characteristics of the respondents while Logistic regression model was used to assess factors that influence respondents’ decision to take up health insurance policy and renew it.

**Results:**

The study results indicate that 61.1% of respondents are currently being enrolled in the NHIS, 23.9% had not renewed their insurance after enrollment and 15% had never enrolled. Reasons cited for non-renewal of insurance included poor service quality (58%), lack of money (49%) and taste of other sources of care (23%). The gender, marital status, religion and perception of health status of respondents significantly influenced their decision to enroll and remain in NHIS.

**Conclusion:**

NHIS has come to stay with clients testifying to its benefits in keeping them strong and healthy. Efforts therefore must be put in by all stakeholders including the community to educate the individuals on the benefits of health insurance to ensure all have optimal access.

## Background

A health system in any country performs instrumental functions of stewardship (oversight), creation of resources (investment and training), delivering services (provision), and financing (collecting, pooling and purchasing) [1]. Ultimately, the effectiveness and efficiency with which these functions are executed determine the extent to which a health system achieves its intrinsic goals of improving health, responding to people's non-medical expectations, and fairness of financial contributions. Ghana has prioritized universal coverage of health care and has therefore put in place policies and programmes to meet this goal. Even though success has been achieved in different aspects of the health sector, health care delivery remains inadequate especially for poor people and other disadvantage groups. The task confronting the health sector remains difficult; life expectancy remains low (60 years), morbidity of preventable diseases remains high; malaria, diarrhoea and other preventable diseases account for about 40% of child mortality, and maternal mortality is still high (240 per 100,000 births) [2].

The challenge since 1981 has been how to find the best combination of public-private partnership that would meet each other part of the way and satisfy the needs and pockets of Ghanaians as well the Government’s finances in the healthcare sector. ‘Cash and Carry’, the system of healthcare financing introduced by the Provisional National Defense Council (PNDC) survived until 2004 when the present health insurance system came into being [3]. Individuals pay premiums in order to receive highly subsidized or even free hospitalization and treatments in future cases. Even then a large number of Ghanaians (about 30 percent) still subsist on cash and carry for their healthcare requirements as they have not registered or subscribed to join the NHIS [3].

According to Olofinnika [4], undoubtedly, insurance is an important mechanism that succors the individuals, states and the nation at large. The story of the National Health Insurance Scheme, since its implementation is that it has come to be accepted by Ghanaians as one of the best social intervention programmes to be introduced in this country. More so because it was not one of those programmes that were sponsored by the Donor Community or the World Bank and the International Monetary Fund (IMF) [3]. To ensure the sustenance of the scheme, membership renewal is one critical factor that cannot be ignored. Insurers want to make it as easy as possible for people to take up health insurance and also renew their policies and it is normal market practice for them to notify policyholders when a policy is about to expire. This is a useful service for many customers. The interest of the study is to understand what determines the individual’s decision to take up National Health Insurance Policy and renew when it expires.

### Health insurance in Ghana

The National Health Insurance Scheme (NHIS) was established under Act 650 of 2003 by the Government of Ghana to provide basic healthcare services to persons resident in the country through mutual and private health insurance schemes. The District Mutual, Private Mutual and Private Commercial Schemes are regulated by the National Health Insurance Council (NHIC) [3]. Funding for healthcare financing under the National Health Insurance Scheme as established by Act 650, comes from a Fund created by the Act, with income from two main sources, also created by the act. These are the National Health Insurance Levy (NHIL), a 2.5 percentage top up of the Value Added Tax (VAT), and a 2.5 percentage transfer from the existing Social Security and National Insurance Trust.

A sketchy overview of health insurance schemes in Ghana shows two forms of schemes that are quite distinctive. These are private insurance companies in the cities and other bigger towns and hospital-based schemes in the districts. The private insurance companies are few and are patronized by employers and few individuals. With the support of development partners various forms of formal health insurance are growing in the districts [5]. At present, quite a number of the districts operate district-based health insurance schemes, which are voluntary and to a greater extent integrated to health care facilities.

### Previous studies on health insurance policy uptake

Several authors discuss the determinants of enrollment such as high cost of premiums, distance to health facilities, place of residence, poor quality of care, timing of premium payments and other behavioral and social factors [6,7]. Even though previous studies by [8,9], have provided ample evidence on determinants of enrollment in MHOs, this has not been looked at with respect to differences between socio-economic groups. However other studies by [10] have shown that there is difference between the rich and the poor with respect to price elasticity of health insurance.

According to Shaw and Ainsworth [11], the choice of a health insurance plan and the extent of involvement by households are driven by two sets of determinants, which are closely related, but are analytically separable - the characteristics of the plan itself, and the personal, household and community characteristics of the individual making the choice. The characteristics of the insurance plans involve the type of medical services offered, the degree of freedom to choose providers and the extent of compensation given [12,13], the quality of care given by the chosen provider and perceived credibility of the insurer [14,15]. Failure to buy insurance among low-risk or high-risk individuals, as it is socially desired, is also influenced by asymmetric information between insured and insurers and plan regulation.

Risk aversion, price sensitivity of medical care and health status of the individual also constitute the personal characteristics underlying the decision to opt for insurance. As indicated by [16], the value of risk spreading increases with risk aversion and variability of medical spending. Additionally, [17] also added that the necessary condition for informal risk sharing schemes to grow is the existence of voluntary exchange. This reciprocity however is sustained if discount rates of people are lower, that is if their degree of relative risk aversion is higher, and the differences between their respective incomes are larger.

## Methods

### Study setting

This quantitative study was conducted in the Volta region of Ghana. It’s capital is Ho. The native and largest ethnic group is the Ewe people and the language spoken is the Ewe language. The Volta Region is located along the southern half of the eastern border of Ghana, which it shares with the Republic of Togo. Greater Accra, Eastern and Brong Ahafo regions share boundaries with it on the west, on the north by the Northern Region, and on the south by the Gulf of Guinea. The region occupies an area of about 20,570 square kilometers or 8.6 per cent of the total land area of Ghana.

Majority of the people are in Agriculture and related occupations. Slightly higher proportions are in Production, Transport and Equipment Operation (13.7%) as in sales work (12.8%). Professional, Technical and Related workers account for 6.3% and Services 3.9%. The percentage of Clerical and related workers in the region is low (2.8%). The region as a whole does not have adequate number of doctors relative to its population. This is underscored by the fact that all the districts have doctor population ratios far above the regional figure (1:21,519), except Ho, which doctor to about 9,800 people [18]. Due to the inaccessibility of Orthodox medical facilities to the rural communities, while traditional healing facilities are within easy reach of between 80.0 to 100.0 per cent of all localities, many people in the region resort to the services of the traditional healers on whom they rely considerably for their primary health care needs. While there is a traditional healer within almost all the localities in the districts, only about 1.4% of the localities in all the 12 districts in the region have a hospital within a walking distance [18].

February 3, 2012 report of the Ghana News Agency (GNA) indicated that a total of 1,470,784 people had subscribed to the membership of the National Health Insurance Scheme in the Region by 31 December 2011 [19]. This represents more than 70 percent of the total population of 2,099,876 base on the 2010 provisional census figure; and the authority hopes to increase its subscribers to about 80 percent by the end of 2012 [19].

### Study population and sample

The target population was defined and restricted to include all adult above 18 years of age within the selected districts. The sample size was determined following [20] as

(1) $$n=\frac{N}{1+N\left(e^{2}\right)}$$

Where *n* is the sample size; *e* = error level (1 – confidence level), and *N* is the estimated total number of adult within the selected districts. Available statistics from the [21] put the overall target population at 12,250. Assuming 95% confidence level, e = 0.05 and a population of 2,250 give a sample size of approximately 300. This was proportionally distributed across selected districts within the region based on number of adult in each district.

Multistage sampling technique was used to select respondents for the study. Firstly, Volta region was purposively selected out of 10 regions in Ghana based on the fact that large proportion of NHIS participants do not renew their insurance when it expired compared with other regions. Secondly, five out of 12 districts in the region were randomly selected for the study. From each of the selected districts, five communities were selected and in each of these communities a simple random technique was used to select the respondents for the study. A number of communities per district vary and it ranges between 13–28. The sample size of 300 was proportionally distributed across the communities using the number of people who have registered for NHIS.

### Data collection and statistical analysis

The instrument used for data collection was questionnaire. This instrument asked for specific factual information concerning the respondents’ uptake of National Health Insurance, their renewal status and personal socio-economic characteristics. The questionnaires were structured with information on age, gender, occupation, education, monthly income, marital status, self perceived health status and perceptions of quality of care and NHIS. The monthly income was estimated based on the primary and secondary occupations as well as revenues from all other cash income including farming produce. Perceptions of quality of care and the NHIS were elicited with a five point Likert scale ranging from “strongly agree (1)” to “strongly disagree (5)”. Insurance status was classified as “never enrolled” (respondents who have never enrolled on NHIS), “previously enrolled” (respondents who were previously enrolled but have not renewed their policy and “currently enrolled” (respondents who are card bearing members of the scheme and are eligible to access services as at the period of the study).

The data was analysed using STATA version 11. Demographics, household characteristics, attitudes and perceptions of respondents on NHIS and quality of care were assessed using descriptive statistics and logistic regression was used to estimate the odds of taken up National Health Insurance Policy and renewing NHIS insurance. The study had 94% response rate.

## Results

### Socio-demographic characteristics of respondents

Majority of the respondents were females representing 66% of total respondents whiles 96 (34%) were males, Table 1. The mean age of the respondents was 36 years and majority was below 34 years. Majority of them were married, 55.6% and eighty-five (30.7%) had basic education whiles 65 (23.5%) had tertiary education. Only 21 (7.1%) had no formal education. The mean income of respondents was GHS 458.70 ($240.73) and 20.5% earned less than GHS 100.00 ($ 52.48) a month whereas only 12.5% earned more than GHS 500.00 ($262.40). Majority of the respondents were Christians whereas 29 (10.4%) were Moslems. Most of the respondents were employed and 79 (29%) were farmers, 52 (19.1%) were civil/public servants and 95 (34.9%) were traders or businessmen. On the respondents perceived health status, 54 (20%) viewed their health status as very good, 63 (23.3%) as good, 91 (33.7%) as fair with only 19 (7.0%) describing their health status as very poor.

**Table 1:** Background characteristics of respondents

**Variable**	**Number of respondents**	**Percentage**
**Sex (n=279)**		
- Female	183	66
- Male	96	34
**Age (n=278)**		
- 18 – 24	55	19.8
- 25 – 34	99	35.6
- 35 – 44	57	20.5
- >44	67	24.1
***Mean = 36 (±0.85)***		
**Marital status (n=279)**		
- Single	97	34.8
- Married	155	55.6
- Divorced	7	2.5
- Widowed	20	7.2
**Educational level (n=277)**		
- None	21	7.9
- Basic	85	30.7
- Secondary	106	38.3
- Tertiary	65	23.5
**Income level (n=244)**		
- <GHS 100	46	20.5
- GHS 100 – 300	88	39.3
- GHS 301 – 500	62	27.7
- >GHS 500	28	12.5
***Mean = 458.7 (± 35.7)***		
**Religion (n=279)**		
- Christian	249	89.3
- Moslem	29	10.4
- Traditional	1	0.4
**Occupation (n=272)**		
- Unemployed	46	16.9
- Farmer	79	29.0
- Civil/public servant	52	19.1
- Trader/businessman	95	34.9
**Perceived health status (n=270)**		
- Very good	54	20.0
- Good	63	23.3
- Fair	91	33.7
- Poor	43	15.9
- Very poor	19	7.0

### Relationship between demographic factors and NHIS policy uptake

As shown in Table 2, gender, marital status, religion and perceived health status had significant association with NHIS policy uptake. Respondents between the ages of 25 and 34 years had the highest percentage (18.2%) of respondent who had never been enrolled. Age of respondents was however not statistically significant (p=0.187) with insured status of respondents involved in the study.

**Table 2:** Socio-demographic characteristics of respondents and its influence on insurance uptake

**Variable**	**Insurance status**			**Chi-square**	
	**Currently enrolled**	**Previously enrolled**	**Never enrolled**		**p-value**
**Age**					
- 18 – 24	28 (50.9)	17 (30.9)	10 (18.2)		
- 25 – 34	60 (60.1)	21 (21.2)	18 (18.18)	8.762	0.187
- 35 – 44	42 (73.7)	12 (21.1)	3 (5.3)		
- >44	40 (59.7)	16 (23.9)	11 (16.4)		
**Gender**					
- Male	45 (46.9)	26 (27.1)	25 (26.0)	16.813	0.003
- Female	102 (55.4)	56 (30.4)	26 (14.1)		
**Marital status**					
- Single	56 (57.7)	22 (22.7)	19 (19.6)	13.267	0.036
- Married	95 (61.3)	42 (27.1)	18 (11.6)		
- Divorced/widowed	19 (70.4)	3 (11.1)	5 (18.5)		
**Educational level**					
- None	14 (66.7)	5 (23.8)	2 (9.5)		
- Basic	55 (64.7)	16 (18.8)	14 (16.5)	4.306	0.635
- Secondary	57 (53.5)	32(30.2)	17 (16.3)		
- Tertiary	41 (63.5)	15 (22.4)	9 (14.1)		
**Income level**					
- <GHS 100	32 (69.6)	10 (21.7)	4 (8.7)		
- GHS 100 – 300	46 (52.3)	23 (26.1)	19 (21.6)	6.401	0.380
- GHS 301 – 500	38 (61.3)	16 (25.8)	8 (12.9)		
- >GHS 500	19 (67.9)	5 (17.9)	4 (14.3)		
**Religion**					
- Christian	155 (62.3)	61 (24.5)	33 (13.3)	9.67	0.046
- Moslem	15 (51.7)	5 (17.2)	9 (31.0)		
**Occupation**					
- Unemployed	26 (56.5)	13 (28.3)	7 (15.2)		
- Farmer	41 (51.9)	38 (48.1)	20 (25.3)	3.417	0.217
- Civil/public servant	22 (42.3)	21 (40.4)	9 (17.3)		
- Trader/businessman	63 (66.3)	21 (22.1)	11 (11.6)		
**Perceived health status**					
- Very good/good	73 (62.4)	29 (24.8)	15 (12.8)		
- Fair	58 (63.7)	20 (22.0)	13 (14.3)	21.931	0.002
- Poor/Very poor	47 (75.8)	10 (16.1)	5 (8.1)		

Among the female respondents, 55.4% were currently enrolled on an insurance policy whiles only 14.1% had never been enrolled as compared to the male respondents (26.0% never been enrolled; p=0.003). About 19.7% of respondents who were single had never been enrolled on insurance and respondents who were divorced or widowed represented the highest percentage of respondents currently enrolled (70.4%). This category of households may be those who see themselves as poor and see the insurance as saviour against out-of-pocket payments and have therefore enrolled.

About 23.8% and 66.7% of respondents with no education were previously and currently enrolled on insurance respectively. Only 9.5% of respondents with no formal education had never been enrolled on any insurance as against 14.1% of respondents with tertiary education. The religious background of respondents also influenced their insurance status with more Christians being currently enrolled than Moslems (62.3 versus 51.7; p=0.046). Finally, perceived health status of respondent statistically influenced insurance status of respondent (p=0.002). About 75.8% of respondent with poor/very poor health status were currently insured or on enrollment as compared to 62.4% of respondents who felt their health status was very good.

### Perceptions about benefits, convenience and cost of NHIS

Table 3 reflects the perception of respondents on the NHIS. About 83% of them generally believed that joining the scheme will benefit them whereas 11% disagreed. Three percent were however not sure if they stand to benefit from the NHIS. Respondents’ views about benefits of the scheme had significant relationship with their insured status (p=0.006). This suggests that respondents made decisions on joining and renewing their insurance based on their perceived benefits of the scheme. Further analysis showed 93% of respondents believed that joining the scheme mean they will not have to borrow money to pay for hospital care. The remaining 7% were either not sure of this position or disagreed. About 81.5% of the respondents also indicated that joining the scheme means that they will save money from paying hospital bills and this had statistically significant relationship with their insured status (p=0.021). This suggests that most people see the NHIS as providing financial protection for subscribers.

**Table 3:** Perceptions about insurance scheme

**Variables**	**Strongly agree**	**Agree**	**Not sure**	**Disagree**	**Strongly disagree**	**F-statistic**	**p-value**
**Benefit of scheme**							
- Joining scheme will benefit me	33.0	50.5	3.0	4.0	7.5	4.254	0.006
- Not need to borrow money to pay hospital care	70.0	23	2.0	7.0	7.5	2.171	0.073
- Will save money from paying hospital bills	60.0	21.5	7.0	3.0	8.5	3.679	0.021
**Convenience of NHIS to subscribers**							
- Collection of NHIS cards is convenient	13.0	8.0	12	29	38.0	3.461	0.046
- Scheme office opening hours is convenient	11	22	9	28	30	1.785	0.121
- Scheme office location is convenient	7	14	8	33	38	2.761	0.092
**Perception of the Price of NHIS**							
- Premium for the package is too high	13	23	5	31	28	5.018	0.001
- Registration fee is too high	21	30	2	42	5.0	4.872	0.012

Outcome variable: insured status.

From the analysis, most respondents do not find the scheme office location convenient in the study area. The breakdown revealed about 38% strongly disagreed, 33% disagreed, whereas only 21% felt it was convenient. The higher perception of the inconvenience of office location is potential disincentive for policy renewal by some subscribers. Again, majority were not comfortable with the scheme office opening hours. This was observed with 11% strongly agreeing, 22% agreeing, 9% were not sure, 28% disagreeing and 30% strongly disagreeing to the statement “*The district scheme opening hours are convenient*”.

Most people also see the period for collection of cards to be a bit laborious. In responding to the statement *“the collection of insurance cards is convenient”*, only 21% agreed while as many as 67% disagreed. Convenience with collection of insurance cards had statistically significant relationship with respondents insurance status indicating that it is an important factor in respondents’ decision to renew insurance cards or not. With respect to the premium for the package, it was generally considered okay by most respondents. This was observed when as many as 59% did not see the premium for the package to be too high, Table 3. The perception about the cost premium packages had statistically significant relationship with respondents decision to enroll and renew their insurance (p=0.001).

Generally people see the price of health insurance to be very high. Specifically, 51% consider the registration fee to be very high while 47% felt it was okay. This suggests that most respondents would prefer a reduction in the cost of registration for the NHIS. This again suggests that the package in its entirety is not seen as being expensive but rather the cost of registration. Respondents decision to enroll and renew NHIS was also based on the registration fee for the insurance (p=0.012).

### Factors influencing NHIS renewal (Multivariate analysis)

Table 4 presents the results of a stepwise logistic regression analysis of the factors influencing the decision to renew health insurance. Model 1 presents results of socio-demographic factors. Model 2 involves socio-demographic characteristics and respondents perceptions about NHIS whiles model 3 involves all factors considered under study.

**Table 4:** Results of logistic regression analysis of factors influence decision to renew NHIS

**Covariates**	**Model 1**	**Model 2**	**Model 3**
	**OR (95% CI)**	**OR (95% CI)**	**OR (95% CI)**
***Socio-demographic characteristics***			
Gender (ref =Male)	2.5 (1.7, 3.8)**	2.3 (1.4, 5.2)*	1.9 (0.9, 6.4)
Marital status (ref = single)	3.1 (1.1, 9.0)*	2.6 (0.6, 5.3)	2.0 (0.3, 9.3)
Religion (ref = Christianity)	0.9 (0.1, 3.1)	0.8 (0.1, 2.5)	0.8 (0.2, 3.3)
Perceived health status			
- Very good/good (ref)	1	1	1
- Fair	0.9 (0.1, 9.2)	0.8 (0.2, 10.1)	0.6 (0.2, 9.9)
- Poor/Very poor	1.7 (1.3, 4.7)*	1.7 (1.2, 5.1)*	1.9 (1.1, 7.3)**
***Perception about NHIS***			
- Joining scheme will benefit me (ref = agree)		0.3 (0.0, 3.7)*	0.4 (0.1, 4.1)
- Will save money from paying hospital bills (ref = agree)		0.7 (0.1, 3.5)	0.6 (0.1, 4.3)
- Collection of NHIS cards is convenient (ref = agree)		0.3 (0.0, 8.1)	0.3 (0.1, 8.5)*
- Premium for the package is too high (ref = agree)		2.5 (1.3, 10.4)**	2.3 (1.2, 9.5)*
- Registration fee is too high (ref = agree)		1.9 (0.5, 5.9)	1.6 (0.1, 7.8)
***Quality of care under NHIS***			
- Quality of drugs is good (ref = agree)			1.1 (0.2, 3.9)
- Staff attitude has improved under NHIS (ref = agree)			0.7 (0.1, 6.2)
Number of obs	275	274	270
LR chi2(4,9,11)	143.46	156.26	164.89
Prob > chi2	0.0000	0.0000	0.0000
Log likelihood	−344.01797	−364.1596	−371.02163
Pseudo R2	0.48025	0.523820	0.648281

Single = single, widowed, divorced. Outcome = renew NHIS insurance. *p<0.05; **p<0.1.

The models gave a McFadden R – squared of about 0.48, 0.52 and 0.64 respectively (Table 4). The log likelihood ratio (LR) statistic is significant at P<0. 001 meaning that at least one of the independent variables included in the model has a coefficient different from zero. Given these two goodness of fit measures, it can be concluded that the logit models used has integrity and is appropriate.

The gender of respondents, marital status and the perceived health status of respondents were socio-demographic characteristics that influenced their decisions to renew NHIS insurance. In model 1, female respondents were significantly 2.5 times more likely to renew their insurance as compared to the male respondents (OR=2.5; p<0.01). This trend was similar in model 2 with odds ratio of 2.3. Respondents who were married were also 3.1 times more likely to renew their health insurance and this relationship was statistically significant (OR=3.1; p<0.05). The odds rations however decreased in models 2 and 3 and did not show significance. Respondents who viewed their health as poor showed increased likelihood of renewing NHIS insurance in all models with odds ratios of 1.7, 1.7 and 1.9 in models 1, 2 and 3 respectively as compared to those who viewed their health status as good.

Respondents’ perceptions about NHIS significantly influenced their decision to renew their insurance. Respondents who disagreed to the assertion that joining the scheme stands to benefit them were less likely to renew their insurance as depicted in model 2 (OR=0.3; p<0.05). Respondents’ decisions to renew health insurance were also based on their convenience about the processes. Respondents who disagreed that the collection of NHIS cards was convenient were also less likely to renew their health insurance (OR=0.3; p<0.05). Premium of the insurance and cost of registering for the insurance also played key role in one’s decision to renew insurance. Controlling for demographic characteristics and perceptions about NHIS, respondents who did not view the insurance premium as high were 2.5 times more likely to renew their insurance (OR=2.5; p<0.01) in model 2, and 2.3 times more likely to their insurance (OR=2.3; p<0.05) in model 3.

## Discussion

### Trend of enrollment in NHIS

Majority of the respondents in this study were currently insured with the respective Mutual Health Insurance Scheme. This constituted 171 respondents (61.1%). Sixty-seven (23.9%) were previously insured with the scheme whiles 42 (15%) have never enrolled in the Health insurance scheme. The level of enrollment was however inconsistent with existing literature that shows low enrollment among the poor to be a problem facing health insurance schemes in low-income countries [22,23] including Ghana [24]. This inconsistency might have been as a result of increase enrollment in the scheme over the years as a result of increasing education of the benefit of the scheme among the populace.

As shown in this study, the attractiveness of the scheme was somewhat a determinant of people’s decision to enroll or not. This was evident s people cited “*scheme not attractive”* as a reason for not enrolling in the scheme. Others also cited lack of money and having alternative sources of care. Respondents who had not renew their insurance also cited poor quality of service at the facility, lack of money to renew insurance and taste of other sources of care. This was consistent with a the study by [25] where respondents who were previously enrolled cited high cost of premiums and lack of confidence in the schemes as the main reasons for not renewing membership. However, as shown by other studies, trust is a sine qua non for enrollment [26,27].

As indicated above, failure to meet clients’ expectation about service quality at the various facilities also contributed to the decision not to renew their insurance. Previous studies reported that demand for health care is sensitive to the quality of service provided and that even poor households limit their demand for health care when the services are poor quality, but are less sensitive to changes in quality of service [28,29]. One major determinant of clients’ confidence in the scheme is the technical arrangements made by the scheme management and this may influence people’s perception of personal benefits. This was evident in this study as respondents cited unattractiveness of the scheme as a reason for never enrolling in the scheme.

### Socio-demographic characteristics and enrollment in NHIS

Results of this study also showed the influence of socio-demographic characteristics in decision to enroll in health insurance schemes. Gender was a significant determinant of one’s insurance status. In the multivariate analysis, females were significantly more likely to renew their health insurance as compared to male respondents. This might be due to the fact that women, representing the more vulnerable in society in terms of health utilization tend to resort to insurance where risk is generally spread and offers some financial protection.

Again, women represent the greatest users of health care and might have enjoyed special recognition under the insurance scheme. Women, as care-givers for children and other sick members of the household, coupled with their vulnerability and physiological make up are likely to have positive attitude towards insurance decisions than do their male counterparts. Previous literature however suggests that health financing schemes do not necessarily bring positive outcomes, for women are at times excluded from the scheme due to high premium rate, power relations within the households, class structure, political and geographical reasons [30]. Again as Wuyts [31] indicated, *‘socially determined structures and processes impede access of some members of society to economic resources, social goods and institution*’ and women and children are often the greatest victims in this case.

Results from this study also indicate the influence of one’s marital status on taking insurance coverage and renewing insurance. This was consistent with results from other studies where married respondents were more likely to take insurance coverage [32,33]. This study however reported no significant association between occupation and enrollment in NHIS and this was inconsistent with studies by [34] and [35] where respondents who were employed were more likely to undertake coverage.

xThe study found significance influence of health status on decision to enroll in the NHIS. This was however inconsistent with the study by [26] which found no evidence that household health status or prior health service utilization influenced enrolment in Community Health Insurance (CHI). Evidence from other sub-Saharan settings [9,36] indicates that the obligation to enroll the entire household can successfully serve to limit adverse selection into a scheme. Investigating the effect of insurance membership among farmers in rural Senegal, Jütting [36] observed that membership bore a strong positive effect on the probability of going to a hospital.

The educational level of respondents did not influence their enrolment into NHIS. This study recorded a high level of respondents with tertiary and secondary school education although data on the study population shows that at the percentage of Clerical and related workers in the region was low. This could indicate that majority of people who enroll in NHIS have higher level of education. There was however no significant association between educational level of clients and enrollment in NHIS in this study.

### Perception about the NHIS scheme

Perceptions about technical processes of the scheme and the benefits one is likely to get from subscribing to the scheme increases the likelihood of enrolling in the insurance scheme. Trust can be enhanced when people see that their preferences matter. When the scheme administrators tend to be responsive to the community’s preference, people’s overall satisfaction with the community scheme’s services is likely to increase. In this study, majority of the respondents agreed that joining the scheme will benefit them and this perception significantly influenced decision to enroll in the NHIS. Other respondents also believed that the scheme offered some form of financial protection in terms of their health care expenditure and this influenced their decision to enroll in the scheme. Consistently, respondents reported similar reason for enrolling in NHIS in the study by [25]. This was supported by evidence from a study in Rwanda which reported that insurance membership has significantly decreased out-of-pocket spending for a full episode of illness for sick members with and without a visit and at the same time has substantially improved members’ access to the modern health care system [9]. The decision to participate in a given health insurance has also been found to be influence by health care expenditure [37].

Respondents in this study expressed their dissatisfaction with technical processes of the scheme. These included collection of NHIS cards, the opening hours of the scheme offices and the location of the scheme. These issues regarding the technical processes has also rendered the scheme unattractive to some people as stated earlier in this discussion. Schneider [38] argues that ‘many MHIS operate within weakly defined legal and political systems; and are based on mutual, non-written agreements that are monitored and enforced by members.’ To him, ‘MHIS managers often lack the technical capacities to manage an insurance scheme and negotiate with providers for better care’.

The price of insurance or premium is another factor influencing the demand for health insurance. In this study, decision to enroll in the NHIS was significantly influenced by perception about the premium package for the insurance and the registration fee. Respondents who disagreed that the premium package is not too high were significantly more likely to renew their NHIS insurance. Affordability of premiums or contributions is often mentioned as one of the main determinants of membership in other studies. For instance in the Nkoranza Scheme in Ghana, the estimated cost of contributions varied from 5% to 10% of annual household budgets [39] and it was recognized that such contributions could be a financial obstacle to membership.

### Perception about quality of care

The quality of services offered by providers under the scheme goes a long way to boost clients’ confidence in the scheme and make the scheme more attractive to prospective clients. Besides, financial incentives created by the insurance providers inferior quality of care may negatively affect MHIS membership. Providing quality health care increases the trust of clients in the health system and insurance in general. In this study, majority of respondents held negative perceptions about the technical quality of care, the adequacy of service delivery including rooms, medical equipment and doctors and the attitude of providers and these negatively influenced their decision to renew NHIS policy. This indicates that after one subscribes to the NHIS, his or her decision to renew the insurance is partly based on the quality of care from health providers. Consistent with this study, lack of quality of care was cited as the most important cause of non-enrolment in the evaluation of the Maliando scheme in Guinea-Conakry [40].

Mladovsky and Mossialos [41] from a ‘health system’ perspective propose that ‘trust decreases the likelihood of adverse selection and moral hazard and increases willingness to pay for healthcare’. These include improving behavior of medical staff to patients, such as increased levels of politeness, improving quality of care (through strategic purchasing); transparency and accountability among those managing the scheme; recourse to justice to punish fraud and increased community participation in the scheme management.

### Limitations

The study however might suffer some methodological limitations. Firstly, the study did not sample the views of NHIS staffs and management which could have been important in looking at all factors influencing enrollment in the scheme. Secondly, the use of quantitative techniques to explore perceptions might not have captured critical in-depth responses and emotions of respondents on how they feel about the NHIS. However, using the Likert scale with varied points helped provide a range that captured the intensity of their feelings for a given item.

## Conclusion

The results showed a positive influence of certain socio-demographic characteristics on the decision to enroll and renew health insurance policy. However, negative perceptions about the NHIS and the quality of care decreased the likelihood of enrolling in the scheme. It can be concluded that, improvement in the technical processes in the scheme management and the quality of care will stimulate voluntary enrollment and renewal rate of the health insurance policy.

The study outcome is consistent with the theoretical framework which indicates that enrollment and renewing a health insurance policy is influenced by scheme factors (convenience, price and benefits), individual factors (gender, religion, marital status, perceived health status) and provider factors (quality of care, staff provider attitude). Ensuring strict policies to make the policy much affordable and improve quality of care will improve enrollment outcome. Better identification of the poor with subsequent provision of premium exemptions will further enhance the realization of the equity goal of the NHIS.

Finally, to enhance renewal and retention of members, the NHIS policy should allow flexibility of premium payments to make insurance more affordable.

## Competing interests

The authors declare that they have no competing interests.

## Authors’ contribution

The study was conceived and designed by DA-V and DB. Both were involved in the data analysis and interpretation of the study findings. Both authors reviewed and critically revised the manuscript for important intellectual content and agreed to submit the manuscript for publication.
